# What is the applicability of tubal ligation with the vaginal natural orifice transluminal endoscopic surgery technique and its value in patient comfort?

**DOI:** 10.1590/1806-9282.20231085

**Published:** 2024-05-17

**Authors:** Süleyman Serkan Karaşin, Ömür Keskin

**Affiliations:** 1 University of Health Sciences, Bursa Yüksek İhtisas Training and Research Hospital, Department of Obstetrics and Gynecology – Bursa, Turkey.

**Keywords:** Natural orifice transluminal endoscopic surgery, Tubal ligation, Laparoscopy, Operative surgical procedures

## Abstract

**OBJECTIVE:**

The aim of this study was to observe the feasibility of the tubal/adnexal approach using vaginal natural orifice transluminal endoscopic surgery and compare its contribution with surgeon ergonomics and postoperative patient comfort with that of conventional laparoscopy.

**METHODS:**

We completed this study retrospectively with 47 patients. Patients were followed at their postoperative first month. We analyzed the usability of the vaginal natural orifice transluminal endoscopic surgery method over conventional laparoscopy by comparing the demographics, surgical data, and postoperative findings collected between the two groups.

**RESULTS:**

Patients in the conventional laparoscopy group were older (39.1±3.3 years) than those in the vaginal natural orifice transluminal endoscopic surgery patient group (p=0.005). Pain intensity 24 h after surgery was lower in the vaginal natural orifice transluminal endoscopic surgery group (p=0.003), while sexual function and dyspareunia did not differ between the two groups in the first month. Patients in the vaginal natural orifice transluminal endoscopic surgery group were more relieved about painlessness and the comfort it brought than the conventional laparoscopy group (p=0.027, χ^2^=12.56).

**CONCLUSION:**

Patients subjected to the vaginal natural orifice transluminal endoscopic surgery procedure showed higher levels of satisfaction, less postoperative pain, and greater comfort than those subjected to conventional laparoscopy.

## INTRODUCTION

Cost-effective methods that emphasize patient comfort and minimize complications in gynecological surgeries are recurrent research areas. Minimally invasive techniques have become common in gynecologic surgery over the past 20–30 years.

Natural orifice transluminal endoscopic surgery (NOTES) is one of the most significant innovations in surgery and gynecology since the advent of laparoscopy^1^. In 2013, Yang et al.^2^ reported on the first transvaginal NOTES adnexectomy. Of the seven cases described in these studies, all were conducted successfully without conversion to the traditional laparoscopic approach.

After this study, vaginal NOTES (v-NOTES) has been increasingly embraced as a minimally invasive modality for various gynecological surgeries, including hysterectomies, myomectomies, and uterosacral ligament suspensions. This procedure may be particularly effective and safe for selected populations, such as obese women or those with large uteri^3,4^.

Despite the positive results, data on v-NOTES need to be expanded. Unknown factors regarding this technique include the ease of application, its effect on the comfort of the surgeon, the impact on the comfort and pain level of the patient after surgery, and its effects on the comfort of sexual activity after the procedure.

Our aims in this study were to observe the feasibility of the tubal/adnexal approach using v-NOTES, compare it with conventional laparoscopy, and examine its contribution and benefits in terms of surgeon ergonomics, operation time, and postoperative patient well-being.

## METHODS

This retrospective study analyzes specific surgeries performed in a third-level hospital during 6 months between August 2022 and January 2023. The Bursa Yüksek İhtisas Training and Research Hospital Ethics Committee approved the applied methods with ethics committee number 2011-KAEK-25 2022/11-18. Informed written consent was obtained from all patients.

### Patient selection

We planned to recruit 47 volunteers in total. The study population included women older than 18 who requested permanent surgical contraception and did not have a Grade 2 or greater prolapse according to the POP-Q system. Patients were excluded from the study if they had pelvic inflammatory disease, suspected malignancy, a history of rectovaginal endometriosis, pregnancy, or a pelvic abscess. In addition, a bilateral salpingectomy was offered instead of tubal ligation to reduce the risk of ovarian cancer.

The study was designed to compare conventional laparoscopy with that of v-NOTES based on patient selection. We recorded the age, parity, body mass index, previous surgery history, whole blood parameters, and modified POP-Q findings, including C, Ba, and Bp points of each patient preoperatively on the study forms.

### Implementation

**Figure 1:**
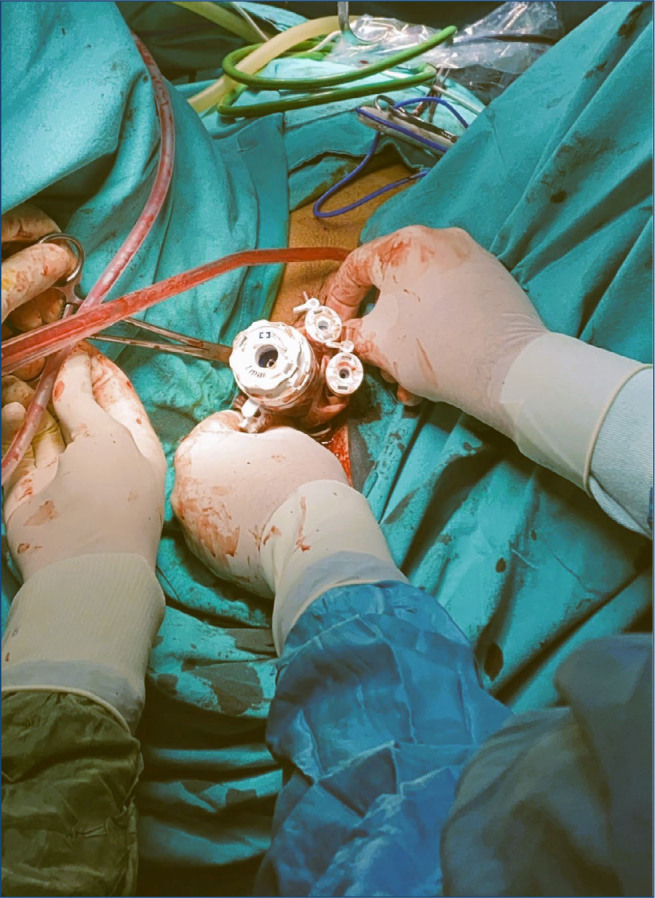
Image of port creation and its position in the patient.

All procedures were performed by surgeons. The co-authors of this study followed previously published surgical techniques regarding the CL and v-NOTES approaches^5,6^. All patients were administered a prophylactic antibiotic treatment consisting of 2 g of intravenous cefazolin 30 min before surgery^6,7^. After general anesthesia and endotracheal intubation, the patients were placed in the Trendelenburg position by lithotomy. A single 2.5-cm incision was made in the posterior vaginal fornix, and the pouch of Douglas was opened to insert the NOTES port. This self-constructed device consisted of a 75-mm silicone vaginal ring (pessary) attached to a size eight powder-free surgical glove. We prevented the glove from opening during the procedure by rolling the ring inside the glove inward twice. One finger of the surgical glove was removed to insert a 10-mm reusable trocar for CO_2_ insufflation and laparoscopic entry. Three 5-mm reusable trocars were inserted through the remaining fingers of the glove to position the reusable laparoscopic instruments (Figure 1). We used standard 30° 10-mm laparoscopy (Karl Storz SE & Co. KG, Tuttlingen, Germany). The reusable conventional laparoscopic instruments utilized were bipolar forceps, atraumatic forceps, and an aspiration-irrigation cannula (ENDOPATHTM Grasper 5 mm, Ethicon, Inc., Somerville, NJ, USA).

With the aid of a Breisky posterior vaginal retractor, we pushed the pessary ring of the NOTES port into the peritoneal cavity and perpendicular to the base of the pouch of Douglas.

### Surgical procedure

An operator controlled the optics during the surgery and manipulated the uterus with the aid of a grasper when required. A second operator conducted the surgery using a bipolar grasper in one hand and a grasper in the other hand. After the ureters were identified, salpingectomy was performed from the fimbria to the uterine horn or ligation with bipolar assistance from two separate areas of the tube. After the procedure, we completed the process by re-visualizing and providing hemostasis with bipolar coagulation if necessary. Then we closed the colpotomy incision with an absorbable polyglactin 1.0 suture in a locked, continuous technique.

### Data analyses

Postoperative patient comfort, postoperative pain, pelvic pain, and sexual function variables were measured. We used the 35-question “multidimensional quality of life scale” questionnaire before surgery and during the first month after surgery to assess patient comfort and satisfaction^8^. The impact of pelvic pain of the patient on the quality of life was assessed using the pelvic pain impact questionnaire (PPIQ)^9^. The assessed domains reflected the energy level, mood, sleep, and gastrointestinal function of the patients, as well as their ability to sit, engage in functional activities and exercise, and wear specific clothing. Sexual discomfort or pain was measured using the female sexual function index-6 (FSFI-6) scale, and we noted a difference between pre-surgery and the first month after surgery. Total scores range from 2 to 30, with lower scores indicating lower sexual function^10^.

Postoperative pain was measured using a Likert-type visual analog score (VAS) after the surgical procedure and 24 h after surgery. VAS scores ranged from 0 (no pain) to 10 (most severe pain)^11^. Additional analgesics were not routinely given to the patients, and we recorded if they were used. In addition, we recorded the level of sexual discomfort or pain during vaginal penetration, the total duration of the surgery (from the first incision to the last suturation, in minutes), the change in hemoglobin levels, and the length of hospital stay over the first month.

## RESULTS

We included 47 patients in our study, with 28 from the CL group and 19 from the v-NOTES group, with mean ages of 36.1±3.2 and 39.1±3.3 years, respectively. The body mass indexes and birth numbers were significantly different for both groups. However, the number of previous abdominal surgeries was significantly higher in the v-NOTES group (p=0.035). The pain measurement VAS score at 24 h after surgery was significantly higher in the CL group than in the v-NOTES group (p=0.003). We did not find a significant difference in postoperative sexual function when comparing the score values in the first month with those of the preoperative and postoperative score reduction values of the FSFI-6 scoring scale (p=0.12 and p=0.08, respectively). We evaluated postoperative comfort and general satisfaction of the patients using the MILQ scale and found that the values in the first month after surgery were lower in the CL group than in the v-NOTES group (p<0.01 and p=0.02, respectively). A detailed analysis of numerical data between the groups is summarized in Table 1.

**Table 1:** Comparison of clinical and demographic characteristics of the groups in terms of abdominal cleaning method.

Parameters	Conventional laparoscopy group (n=28)	v-NOTES group (n=19)	p
	Median (min–max)/mean±SD	Median (min–max)/mean±SD	
Age (years)*	36.1±3.2	39.1±3.3	0.005
Body mass index (kg/m^2^)*	26.2±4.8	23.7±3.7	0.05
Number of births^#^	3 (2–6)	4 (3–6)	0.07
Previous abdominal surgery^#^	0 (0–3)	1 (0–3)	0.035
24th hour VAS score^#^	4 (1–8)	3 (1–4)	0.003
Duration of surgery (min)^#^	27 (15–45)	22 (15–40)	0.06
Hemoglobin decrease (mg/dL)*	1.4±0.6	1.2±0.4	0.12
Hospitalization (days)^#^	1 (1–2)	1 (1–1)	0.95
Postoperative FSFI-6 score*	13.4±7	16.4±5.8	0.12
Postoperative MILQ score*	196.3±26.2	217.6±10	<0.01
FSFI-6 decrease^#^	4 (0–13)	3 (0–10)	0.08
MILQ decrease^#^	11 (3–26)	6 (2–18)	0.02
PPIQ score^#^	5 (1–15)	4 (2–10)	0.11

Student’s t-test

^*^p<0.05 and Mann-Whitney U test

^#^p<0.05 significant. min: minimum; max: maximum; SD: standard deviation.

Patients in the v-NOTES group were significantly more satisfied regarding the lack of pain and related comfort level than those of the CL group (p=0.027, χ^2^=12.56). Five patients in the v-NOTES group expressed themselves as “very satisfied,” whereas two patients in the CL group stated that they were not satisfied. The number of patients expressing the “I am happy” and “very happy” categories was 13 (46%) in the CL group and 13 (68.4%) in the v-NOTES group. The complete analysis is summarized in Table 2.

**Table 2:** Cross-square analysis table of the groups according to the results of the seventh subtitle parameter of the multidimensional quality of life scale.

Among the groups	Are you satisfied with your pain-free state?				
	Conventional laparoscopy group (n=28)	v-NOTES group (n=19)	n	χ^2^	p
Not satisfied at all	2 (7.1%)	0 (0%)	2 (4.3%)	12.569	0.027
Partially dissatisfied	1 (3.6%)	2 (10.5%)	3 (6.4%)		
Neither satisfied nor not satisfied	4 (14.3%)	0 (0%)	4 (8.5%)		
Partially satisfied	8 (28.6%)	4 (21.1%)	12 (25.5%)		
Satisfied	13 (46.4%)	8 (42.1%)	21 (44.7%)		
Very satisfied	0 (0%)	5 (26.3%)	5 (10.6%)		
**Among the groups**	**How often did you experience discomfort or pain during vaginal penetration?**				
	**Conventional laparoscopy group (n=24)**	**v-NOTES group (n=14)**	**n**	**χ** ^**2**^	**p**
Sometimes pain	1 (4.2%)	0 (0%)	1 (2.6%)	0.616	0.735
Rarely pain	12 (50%)	7 (50%)	19 (50%)		
Never pain	11 (45.8%)	7 (50%)	18 (47.4%)		

Pearson chi-square, p<0.05 was considered significant.

Four patients from the CL group and five from the v-NOTES group did not have sexual intercourse within 1 month. There was no significant difference between the two groups in pain or discomfort during penetration according to the sixth sub-item of the FSFI-6 scale (p=0.735, χ^2^=0.616). Eleven patients (45.8%) from the Cl group and seven patients (50%) from the v-NOTES group stated that they did not feel any pain during this activity (Table 2).

## DISCUSSION

In this study, we documented the feasibility of the v-NOTES procedure in patients requesting permanent contraception through salpingectomy or tubal ligation. Patients who underwent the v-NOTES procedure showed greater satisfaction, less postoperative pain, and a higher level of comfort than those who underwent CL.

There is considerable interest in new methods of minimally invasive surgery, which has led to the emergence of a new field of gynecological surgery, NOTES. Potential benefits of natural orifice surgery in gynecology include less operative pain, shorter hospital stay, lack of abdominal incisions, improved visibility, and extensive lysis of adhesion circumvention to reach the pelvic cavity. Transvaginal access for NOTES is the safest and most feasible of all of the procedures for clinical application. However, the opening of the posterior vaginal space (culdotomy) should be carefully performed to avoid potential complications, especially in the pouch of Douglas obliteration cases^12,13^.

Vaginal surgery may have benefits over other techniques for benign gynecological diseases. However, inadequate visualization of the surgical field and difficulty in accessing adnexal structures in a non-prolapsed uterus restrict the use of transvaginal opportunistic bilateral salpingectomy^7^. Data support the usefulness of v-NOTES in the surgical treatment of adnexal pathologies^14^. v-NOTES can reduce the challenges and potential limitations of vaginal surgery and increase the indications for conventional vaginal surgery^12,13,15^.

Li et al.^5^ published a systematic review on the role of v-NOTES in gynecological surgery in 2019. The current review compared conventional laparoscopy with v-NOTES in two studies and demonstrated that v-NOTES can deliver superior outcomes in terms of blood loss, operative time, and length of stay^16,17^. This review stated that younger patients were more concerned than older patients regarding the v-NOTES procedure, which may be primarily related to anxieties relating to possible sexual repercussions. In this review, postoperative pain was reduced in patients who received the v-NOTES procedure compared with those undergoing the CL treatment. This review concluded that the studied gynecological surgical procedures could be performed safely and consistently by experienced surgeons.

Laganà et al.^12^ compared patients who had undergone salpingectomies with those subjected to v-NOTES and CL in 2021. Patients in the v-NOTES group reflected less postoperative pain and more comfortable surgical outcomes; therefore, that study indicated that the v-NOTES procedure could be a suitable alternative to CL.

In our study, we showed that patients felt less pain after v-NOTES. Theoretically, a v-NOTES incision in the vagina would be expected to result in less pain than that of a skin incision. In addition, the pain and comfort levels in the first month after surgery were more positive in the v-NOTES patient group than the CL group, although this may partly relate to preconceptions of patients regarding surgery. The absence of a fascia incision can explain this situation, although it may be a psychological factor related to the lack of a surgical scar. Nevertheless, more data are required in the literature comparing the two surgical procedures to provide additional explanatory reasons for this situation in the coming years.

In this study, the results regarding postoperative sexual life and dyspareunia were similar between the patient groups. The group that chose CL from the two procedures was younger than the group that chose transvaginal surgery. We think that this may be a reflection of concerns concerning sexuality. Although one of the most severe concerns about transvaginal surgery is sexual dysfunction, we found no signs of withdrawal from coitus or pain in our patient group.

The main limitation of this study was the number of patients. We used 30-degree optics as a standard for visualization; however, using an image with angleless optics and determining its effects on the duration of the surgery could be a future option.

Safe and optimal peritoneal access is critical to performing a v-NOTES procedure. Consideration of the importance of patient comfort and recovery speed will increase as technologies are developed. v-NOTES is a candidate for a primary minimally invasive surgical procedure in the coming years.
